# An impediment to random walk: trehalose microenvironment drives preferential endocytic uptake of plasmonic nanoparticles†
†Electronic supplementary information (ESI) available: Cell viability WST-1 test in presence of glucose, SERS band assignments and UV and TEM characterization of Ag nanoparticles. See DOI: 10.1039/c6sc00510a


**DOI:** 10.1039/c6sc00510a

**Published:** 2016-02-23

**Authors:** Soumik Siddhanta, Chao Zheng, Chandrabhas Narayana, Ishan Barman

**Affiliations:** a Department of Mechanical Engineering , Johns Hopkins University , Baltimore , MD 21218 , USA . Email: ibarman@jhu.edu; b Light Scattering Laboratory , Chemistry & Physics of Materials Unit , Jawaharlal Nehru Centre for Advanced Scientific Research , Jakkur P.O. , Bangalore 560 064 , India; c Department of Oncology , Johns Hopkins University Baltimore , MD 21287 , USA

## Abstract

Developing effective theranostic nanoplex platforms for personalized disease treatment necessitates an understanding of and the ability to control live cell–nanoparticle interactions. However, aggregation of nanoparticles on the cell surface and their subsequent internalization is sparsely understood and adversely impact cellular recognition and viability. Here we report a facile method of precisely modulating the aggregation and uptake for silver nanoparticles without altering their surface geometry or functionalization. Exploiting the stabilization properties of trehalose, our approach enables uptake of nanoparticles while reducing aggregation on cell surface and maintaining cell viability. Electron microscopy reveals the larger utilization of endosomal structures in the trehalose-rich environment compared to the nanoparticles' “free” cytosolic diffusion patterns in the control group. Additionally, in the presence of trehalose, plasmon-enhanced Raman spectroscopy confirms the preservation of the protein structure in the vicinity of the nanoparticles reinforcing the promise of the proposed route for label-free, multiplexed intracellular monitoring.

## Abstract 2

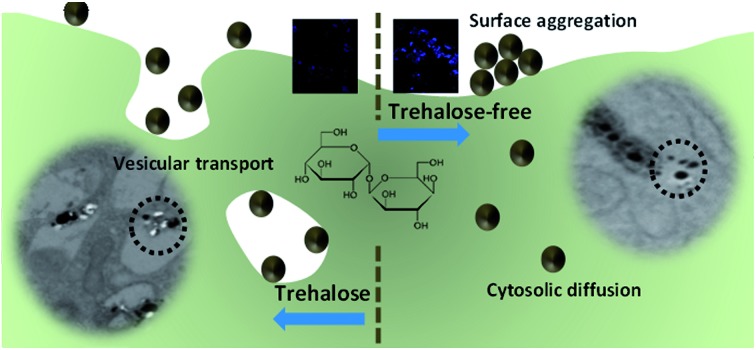
Trehalose changes the mode of internalization of plasmonic nanoparticles predominantly from cytosolic diffusion to vesicular transport maintaining cell viability and reducing membrane-focused aggregation.

## Introduction

The interaction of nanoparticles (NP) with biological entities, especially on the cellular scale, is of great importance in emerging delivery, diagnostic and therapeutic agents for nanomedicine platforms.^1^ For example, devising a reliable palette of NP probes is critical for elucidating fundamental biological pathways, tracking disease progression and monitoring therapy response.^2^ While its application in imaging of multiple species and local chemical environments in the cellular milieu has received considerable attention, limited understanding of the interactions between NP and live cells has often translated to an uncertain cellular uptake and intracellular fate. Recent reports have shed light on the complexity of these interactions due to non-specific associations of the NP surface with a variety of molecular entities that result in a biomolecular corona.^3^,^4^ Because of these interactions, the control over the colloidal stability is often compromised that leads to undesirable aggregation and localization of NP.^5^

Additionally, the surface of most nanoscale imaging platforms, such as quantum dots, carbon nanotubes and plasmonic contrast agents, are functionalized with antibodies, peptides or aptamers to enable recognition of specific cell surface receptors.^6^,^7^ However, the appearance of the corona alters the physico-chemical properties of the NP adversely influencing cellular recognition and targeting specificity.^8^,^9^ The onus is, therefore, to gain a complete understanding of what the cell “sees” when exposed to a NP-containing environment.

Specifically, in order to suppress the non-specific adsorption, researchers have sought to functionalize the NP surface with the antifouling polymer polyethylene glycol (PEG).^10^ Nevertheless, the effect of PEG backfilling on abating the loss of specificity due to corona formation is unclear.^11^ PEGylation, and similar routes, also force a tradeoff between desired NP properties (recognition, uptake, and reduced toxicity) and sensitivity of plasmon-enhanced spectroscopic sensing methods. This is particularly evident for surface-selective approaches, such as surface-enhanced Raman spectroscopy (SERS),^12^ where the proximal presence of noble metal NP and the organic molecule of interest is key. Significantly, the (variable) local aggregation state of the functionalized NPs hinders controlled enhancement of spectroscopic signals from live cells.^13^ Thus, there is a substantive need to design suitable conditions for plasmonic NP–cell interaction and internalization that address the NP surface composition and aggregation behavior in the cellular environment.

Here we report a facile osmolyte-based approach to reduce surface aggregation of plasmonic NPs on the plasma membrane of the cells without resorting to specific surface functionalization. Specifically, our approach builds on the remarkable stabilization properties of trehalose and also affords substantive protection for the cell from NP-induced stresses. We reasoned that trehalose would provide an ideal bioprotectant, as it can stabilize lipid bilayers as well as proteins stemming from a combination of its vitrification, preferential exclusion and water replacement actions.^14^ Important clues also come from our recent observations of trehalose mediated stabilization of lysozyme, which is otherwise known to denature upon interaction with silver NP.^15^ While there are different complementary aspects to NP behavior within the cellular milieu, some of which have been explored in the literature,^16^–^18^ our current work is focused on the role of osmolyte-induced changes in interactions of the NP and biomolecular constituents of the cell, and its reflection on spectroscopic measurements.

Notably, our investigations here reveal that the reduction of NP aggregation and the maintenance of cell viability occur without greatly affecting the internalization of the plasmonic NP in the cells. This technique, therefore, provides a superior route for ultrasensitive, label-free and multiplexed SERS sensing inside live cells due to the direct and close interaction between the nanoparticle and the probed molecules. To obtain a comprehensive understanding of the process of plasmonic NP internalization, we further performed electron microscopy of prostate cancer cells incubated with silver NP in the presence and absence of trehalose. Our transmission electron microscopy (TEM) findings shed new light on the unique modulation of cellular entry in this carbohydrate medium. We also find that the NP–cell interactions in the presence/absence of trehalose have a strong correspondence to the acquired SERS spectra, which independently confirm our hypothesis of the protective role of trehalose at the cellular level.

## Results and discussion

### Investigation of nanoparticle-induced cell viability changes in trehalose microenvironment

**Fig. 1:**
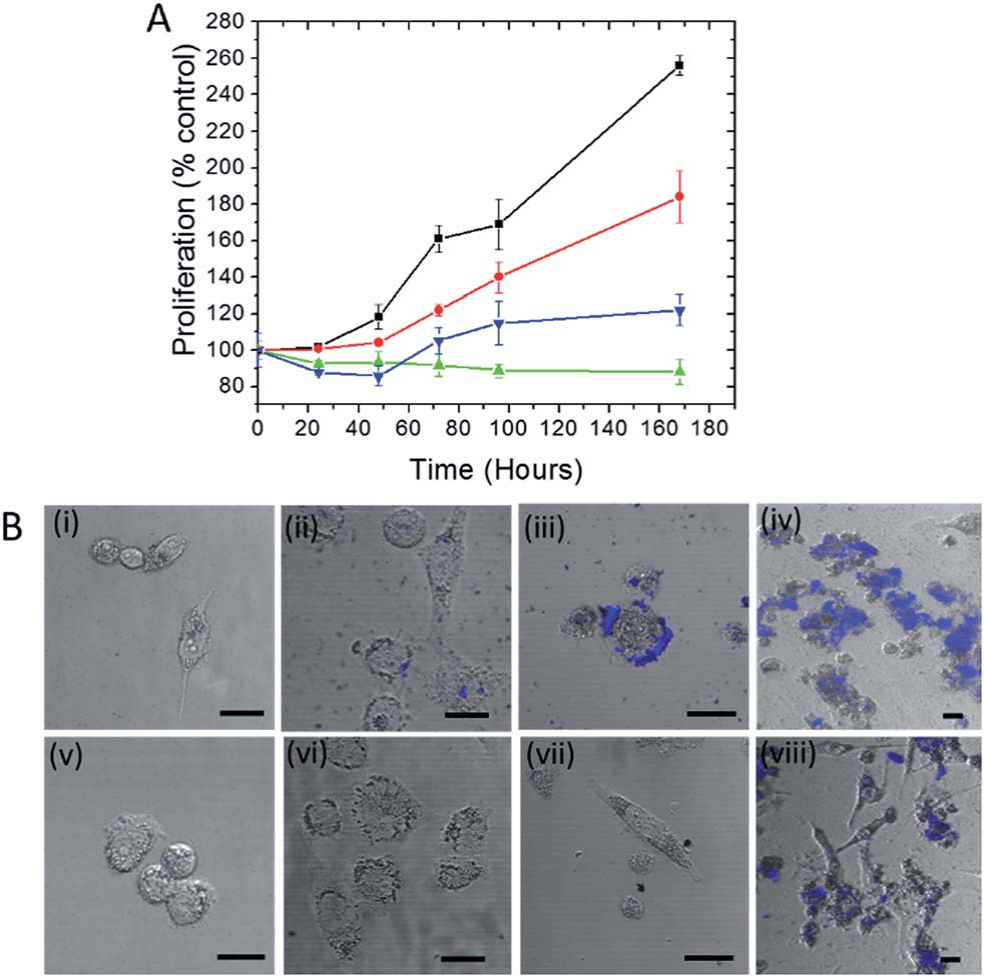
(A) Plot of cell proliferation, as determined by dehydrogenase activity (WST-1 test), of human prostate cancer PC3-Flu cells. The assays were performed with: control cells (black), cells incubated in culture media with addition of trehalose (red), cells incubated with nanoparticles in the presence of trehalose (blue) and cells incubated with nanoparticles in the absence of trehalose (green). (B) Far red fluorescence images overlaid with bright field photomicrographs of cells incubated with silver nanoparticles (i)–(iv) and silver nanoparticles along with trehalose (v)–(viii) for a period of 0, 2, 4 and 7 days respectively. The scale bar represents 10 μm.

Trehalose is a non-reducing disaccharide that has been found to provide exceptional stability to proteins preventing their denaturation from thermal, dehydration and NP-induced stresses.^15^,^19^ We first investigated whether this influence extends to the protection of live cells, where the complexity of NP interactions is significantly higher. Here we selected silver NP as the plasmonic agent because of its greater SERS enhancement in comparison to gold^20^ (and therefore its better suitability in constructing ultrasensitive probes) as well as its higher toxicity to cells.^21^ The WST-1 assay, which determines cell viability by analyzing its metabolic activity, was employed to assess the number of viable human prostate cancer PC3-Flu cells in the presence of silver nanoparticles and in the absence and presence of trehalose for a period of 7 days (Fig. 1A). Clearly, the cell proliferation in the presence of the nanoparticles (green line) is much lesser than the control (without silver nanoparticles) (black line). Indeed, in the former case, the number of viable cells is actually reduced. In contrast, when nanoparticle is added to the trehalose infused cell culture media, the cell proliferation (blue line) – while not at the levels observed in standard cell culture media – is substantially greater than that in the presence of nanoparticles alone. This reinforces our conjecture of the bioprotective role of trehalose in maintaining cell viability.

A particular point of interest, in this regard, is the trehalose-mediated inhibition of cell surface receptor denaturation, which is one of the principal causes of NP-induced membranolysis and cell apoptosis.^22^ Although the negatively charged citrate coated silver nanoparticles are less effective in entering the cell than the positively coated ones, they can potentially disrupt the surface protein receptors as well as the lipid bilayer membrane causing cell death.^23^ Other reasons for NP-induced toxicity include subsequent denaturation or aggregation of proteins inside the cell.^24^ The leaching of Ag^+^ ions from the NP into the cells may also contribute to the toxicity.

In Fig. 1A, the cell proliferation in the presence of trehalose alone (red line) also shows a dip from that observed in standard culture media, which can be attributed to the perturbation of the osmotic balance due to extraneous addition of trehalose. A WST-1 assay in the presence of an alternate carbohydrate (glucose) solution was also performed and the results reveal the reduction of cell viability even in the absence of silver NP (see Fig. S1†). The inhibition of cell proliferation in this case is the result of adverse interactions of glucose with several protein pathways,^25^ some of which may be common with the routes of cell–NP interactions.^26^ More importantly, the detrimental impact of glucose puts into perspective the surprising efficacy of trehalose in the prevention of NP-induced toxicity.

The disruption of the plasma membrane of the cell is directly related to and can be visualized by the local aggregation of nanoparticles on the cell membrane. Transmembrane proteins form an integral part of these phospholipid bilayer membranes. The PC3-Flu cells used in our studies lack bulky transmembrane cancer marker proteins, notably prostate-specific membrane antigen (PSMA) unlike its phenotypically similar counterpart (PC3-PIP).^27^ Here the nanoparticle binding and subsequent aggregation can be largely attributed to the integral plasma membrane proteins. It has been previously reported that the nanoparticles can also disrupt the lipid raft micro-domains to interact with these proteins.^28^ To gain further insights into the process, we visualized the NP aggregation patterns using confocal fluorescence and bright field microscopy (Fig. 1B). The aggregation behavior was observed over a period of 7 days with the size of aggregates, expectedly, increasing with time. The aggregates were mostly concentrated on the surface of the cell and exhibited far-red fluorescence when excited with a 488 nm laser. The latter arises from the interaction of surface plasmons (dipolar as well as multipolar in case of aggregates) with molecules on the cell surface and cytosol, which produces strong fluorescence signals in the range of 700–800 nm.^29^ The progression of the aggregate formation coincides with the decrease in cell proliferation in the presence of silver NP and confirms the profound effect of aggregation on cell membrane disruption and, therefore, on cell viability. In the presence of trehalose, however, the aggregation and the cluster size observed through fluorescence were considerably reduced. Taken together with the WST-1 assay findings, these results demonstrate that trehalose inhibits aggregation of NP on the cell surface, which has a major impact in reducing overall NP toxicity to the cells.

### Interplay of endocytic uptake and cytosolic diffusion of nanoparticles

**Fig. 2:**
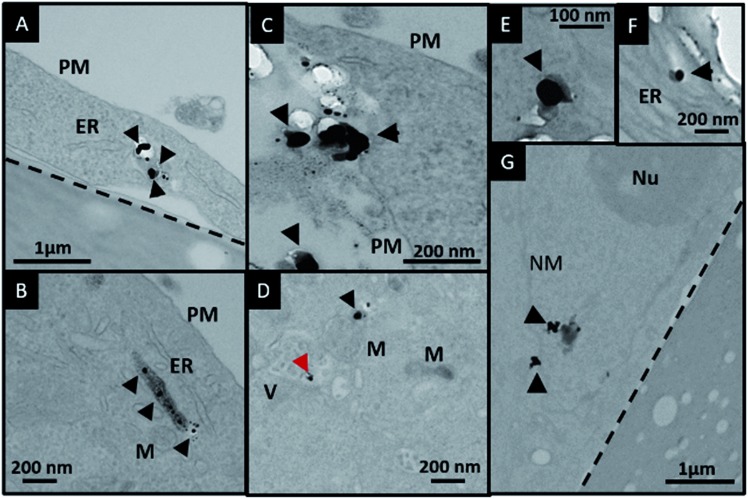
(A)–(G) Localization of silver nanoparticles in PC3-Flu cells in the absence of trehalose in the cell culture media. The black arrowheads indicate localization of nanoparticles in the cytosol and the red arrowhead indicates localization in vesicles. The dotted line demarcates the cell from the underlying substrate. Abbreviations: PM, plasma membrane; ER, endoplasmic reticulum; M, mitochondria; V, vesicle; NM, nuclear membrane; Nu, nucleolus.

**Fig. 3:**
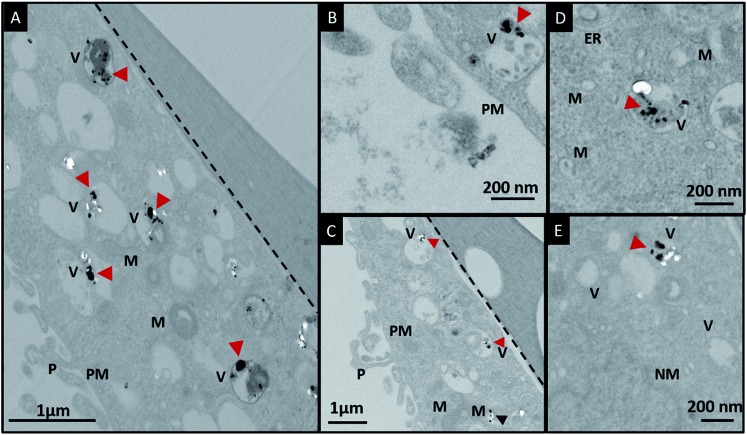
(A)–(E) Localization of silver nanoparticles in cells in the presence of trehalose in the cell culture media. The red arrowheads indicate localization of nanoparticles in the endosomes and the black arrowhead indicates localization in cytosol. The black dotted lines demarcates the cells from the substrate. Abbreviations: PM, plasma membrane; ER, endoplasmic reticulum; M, mitochondria; V, vesicle; NM, nuclear membrane.

**Fig. 4:**
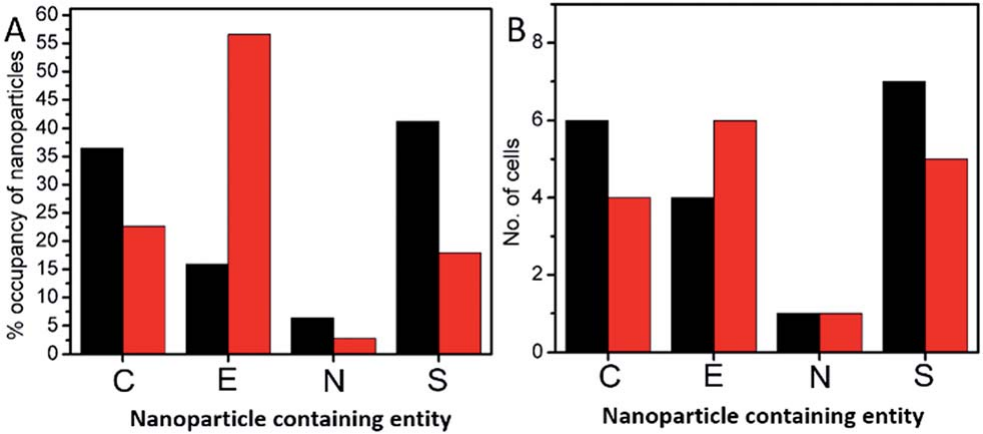
(A) Bar graph of the percentage occupancy of nanoparticles internalized in the PC3-Flu cells in cytosol (C), endosomes (E), nucleus (N) and aggregated at the plasma membrane surface (S). (B) Bar graph of the number of cells in which nanoparticles are found in each of the different components: cytosol (C), endosomes (E), nucleus (N) and plasma membrane surface (S). The black and the red bars correspond to the absence and presence of trehalose environment in the cell culture media, respectively.

In order to investigate the downstream fate of the nanoparticles after uptake by the cells and the impact of trehalose therein, we performed transmission electron microscopy (TEM) on fixed PC3-Flu cells (Fig. 2 and 3). The NPs were internalized into intact cells in both cases (with and without trehalose) without significant difference (Fig. 4B). The size distribution of the just synthesized and internalized nanoparticles, obtained from TEM analysis, is provided in Fig. S3.† The presence of a few larger aggregates indicates agglomeration of the nanoparticles inside the cell, which is less prevalent in the presence of trehalose resulting in a narrower size distribution. However, the major difference observed in case of the trehalose environment is the presence of the NP aggregates in well-defined endosomes (*ca.* 57% as opposed to 16% in the absence of trehalose) (Fig. 4A). Evidently, in case of cells incubated with silver nanoparticles in the absence of trehalose, the nanoparticles were localized mostly in the cytosol. This provides compelling evidence of the influence of trehalose on the modes of internalization of the NP. On the other hand, the localization of NP aggregates inside endosomes indicates that the preferred mode of uptake is endocytosis (Fig. 3).^30^ The clathrin or caveolae mediated endocytic uptake involves cell surface receptors and is the most common way for internalizing nanoscale objects including NP and viruses.^30^ The presence of trehalose minimizes the toxic effect and aggregation on the cell surface *via* internalization through the clathrin-coated pits. While other routes of internalization to cytosol are known, it has previously been observed that endocytosed NP are mostly confined to endosomes.^11^ This reduces the chance of NP to interact adversely with the cytosolic proteins as well as to undergo retrograde transport to the Golgi apparatus and endoplasmic reticulum. It has been reported that trehalose uptake in cells happen through a clathrin dependent endocytic mechanism.^31^ Due to the decrease in protein interaction in presence of trehalose as well as increased endocytic uptake of trehalose, one can reasonably infer that the probability of the nanoparticles to be entrapped into these endosomes increases in this environment. Conversely, in the absence of trehalose, the NP diffuse directly into the cytosol (Fig. 2) and are not localized into endosomes. These NP appear to strongly interact with the surface of the cells and escape the endocytic pathway. Rather, diffusion is prevalent when the nanoparticles disrupt the plasma membrane, which has also been suggested by molecular dynamics simulations.^28^ Few nanoparticles are found to cluster in the Golgi apparatus and ER as well as in the nucleus (Fig. 4A) although most of the localization happens in the endosomes and the cytosol (Fig. 4B). The cell surface aggregates seen in the fluorescence imaging experiments can also be observed in the TEM images, though some of these aggregates may have been transported there through exocytosis. Also, the aggregate formation seen on the cell surface in the presence of trehalose is lesser than that in the absence of trehalose. We propose that since the diffused NPs are exposed to cytosolic proteins, they can readily intrude into various cellular pathways thereby having a deleterious effect on cell viability, as previously confirmed from the WST assay measurements.

### Viewing cell–nanoparticle interactions through SERS measurements

Finally, we performed SERS measurements to elucidate NP-induced alterations to the native state of the cellular components by analyzing their vibrational modes. Specifically, the plasmonic nanoparticles interact with the incident photons generating localized surface plasmon resonance, which results in intense electromagnetic fields in the vicinity of the nanoparticles.^32^ This concentrated electric field in a small volume around the nanoparticle makes the SERS measurements surface selective and provides insights into the varied paths the nanoparticles traverse. Although designer fluorescent nanoparticles have been extensively used to track their motion inside cells,^33^ the intrinsic cellular microenvironment (and any changes induced by the uptake of nanoparticles) is best probed using direct, label-free approaches. As shown by Ando *et al.*, SERS can be advantageously used to track the cellular transport pathway in real time using non-labeled gold nanoparticles.^34^ It has also been reported recently that the vibrational information obtained is greatly influenced by the composition of the corona formed around the nanoparticle inside the cell.^35^ Yet, understanding the complexities of nanoparticle transport, their impact on the cell viability and the influence of different microenvironments is still limited. Here, we employ SERS featuring non-labeled silver nanoparticles to not only offer a snapshot of the internalized nanoparticles inside the cell but to also enable the analysis of the molecular corona constituents depending on the transport pathway.

**Fig. 5:**
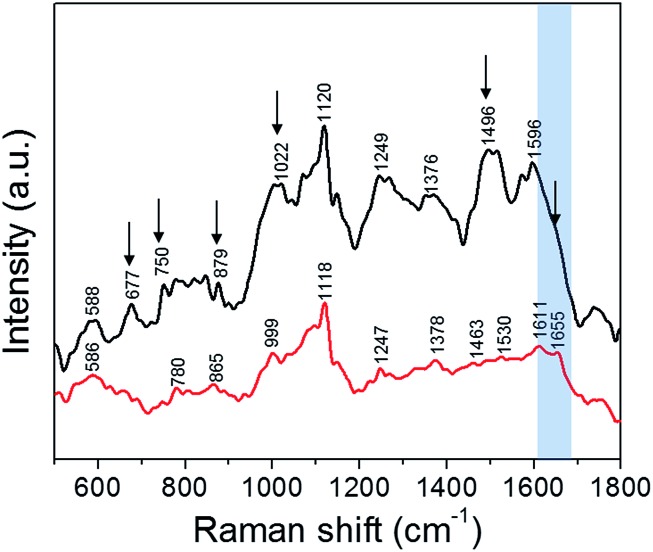
SERS spectra collected, using a 532 nm laser source, from PC3-Flu cells incubated with silver nanoparticles: in the absence of trehalose (black); and in the presence of trehalose (red). The spectral changes are indicated by black arrows and the amide I region is highlighted in blue.

We studied live cells in solution phase to characterize the molecules that interact with the silver NPs following the latter's cellular uptake (Fig. 5). These vibrational fingerprints inform on the interaction in the immediate endosomal and/or cytosolic environment, as SERS selectively probes the localized nanoscopic volume surrounding the plasmonic NP.^36^ The SERS spectra were dominated by vibrational features characteristic of phospholipids (586, 780, 879, 1376 and 1463 cm^–1^), carbohydrates (1022, 1496 cm^–1^), amino acids (750, 879, 999 and 1596 cm^–1^), proteins (1247, 1530 and 1655 cm^–1^) and nucleic acids (677, 780 and 1120 cm^–1^) (refer to Table S1† for a complete list).^37^ One of the most distinct SERS modes observed in live cells is the 1120 cm^–1^ line, which corresponds to the O–P–O DNA backbone vibration.^36^ This phosphate mode arises from the accumulation of the digested nucleic acids in the lysosomes, which is also reported as a key site for nanoparticle accumulation.^38^ The band observed at 999 cm^–1^ corresponds to phenylalanine whereas the DNA bases vibrational modes occur at *ca.* 670 cm^–1^.^36^

Perhaps, most notable in the spectral profile is the amide modes, which are a combination of C=O stretch and N–H bend vibrations. The amide transitions of protein backbones are intrinsic to all proteins and their spectral positions are directly determined by the type of secondary structure (conformation).^36^ We observe that the amide-I mode at *ca.* 1655 cm^–1^ corresponding to the α-helical conformation of the polypeptide backbone is prominent in presence of trehalose but becomes indistinct in absence of trehalose – a clear indication of the disruption of protein structure. The overall decrease in SERS intensity in presence of trehalose also indicates reduced interaction of the NP with the cytosolic or membrane components due to the NP localization in the endosomes. This is consistent with our previous work on interaction of silver nanoparticles with the protein lysozyme where it was seen that the presence of trehalose minimizes the nanoparticle–protein interactions.^15^ The reduction in SERS signal intensity is also an indication of the minimized interaction of the nanoparticles with the intracellular biomolecules. In other words, the NP uptake in the presence of trehalose provides a direct route to measurement of the endosomal constituents including the vesicular membrane, whereas in its absence the SERS readout reflects the collective protein denaturation in the plasma membrane and the cytosol. The latter phenomenon is responsible for apoptosis when the cells are exposed to bare NP. The significant improvement of cell viability in the trehalose microenvironment should facilitate the use of SERS over an extended time frame to study complex cellular processes *in situ* including the effects of drugs and progression of pathological conditions.

## Conclusions

In summary, we have proposed and demonstrated an alternative non-functionalization route for cellular internalization of silver NP without significantly compromising the cell viability. The exceptional protein stabilizing effect of trehalose is translated to live cells where the cell surface entities are preserved from the NP-induced stress. Using a combination of molecular spectroscopy and imaging, we have further elucidated the mechanisms of NP–cell interaction and subsequent uptake in the presence/absence of trehalose stabilization. As silver NPs offer strong foundations for plasmon-enhanced spectroscopic measurements, our present work also provides a novel route to ultrasensitive, multiplexed and label-free characterization of the endocytosis process.

## Experimental section

### Materials

Trehalose dihydrate, silver nitrate and sodium citrate were obtained from Sigma Aldrich and used without further purification. Potassium hydrogen phosphate and potassium dihydrogen phosphate (Sigma Aldrich) were used to prepare buffer solutions using ultrapure Milli-Q water.

### Cell culture

Human prostate cancer PC3 cells transfected with the plasmid alone (PC3-Flu) were obtained from Dr Martin G. Pomper (Johns Hopkins school of Medicine, Baltimore, MD). Cells were maintained in RPMI 1640 (Sigma Aldrich) supplemented with 10% fetal bovine serum (Sigma Aldrich) and 1% penicillin–streptomycin (Sigma Aldrich) in a humidified incubator at 37 °C/5% CO_2_.

### Preparation of silver nanoparticles

Colloidal silver nanoparticles were synthesized by the following the standard Lee–Meisel^39^ method using sodium citrate as a reducing as well as capping agent. 18 mg of silver nitrate was dissolved in 100 ml ultrapure water and brought to boiling point. Subsequently, 2 ml of 1% sodium citrate solution was added to the boiling solution and stirred for 60 min. After the heating was turned off, the solution was brought back to room temperature by constant stirring. The nanoparticles were characterized by transmission electron microscope, UV-visible spectroscopy (Fig. S2†) and dynamic light scattering (DLS) studies (Fig. S3A†).

### Confocal fluorescence microscopy

The confocal fluorescence microscope LSM 510 META scanning confocal microscope with a Plan Apochromat 63× oil-immersion lens (NA = 1.4) (Zeiss, Germany) was used. The microscope was controlled using Zeiss LSM acquisition software. The solution containing the cells incubated with the silver nanoparticles was placed on a quartz bottom Petridish for imaging in an inverted geometry. An argon laser with excitation wavelength of 488 nm was used for imaging.

### Transmission electron microscopy

The cells were incubated with silver nanoparticles for 3 hours and the samples for TEM were prepared according to the procedure reported previously.^40^ Serial sections of the fixed sample was cut and mounted on copper grids. A Philips EM 410 transmission electron microscope (FEI) was used to capture images with a FEI Eagle 2K camera.

### WST-1 cell proliferation assay

Viability of cells treated with silver nanoparticles was measured using by the Cell Proliferation Reagent WST-1 (Sigma Aldrich) in accordance with the manufacturer's instructions. Briefly, PC3-PIP and PC3-FLU cells (2 × 10^3^ cells per well) were cultured in 96 well plate of flat bottom in a final volume of 100 μl per well culture medium. The silver nanoparticle concentration was maintained at 0.5 nM at each well. At the trehalose containing wells, the concentration of trehalose was maintained at 0.75 M. At the indicated time points, 10 μl of this reagent was added to each well and incubated for an additional 1 h. Then, the plate was shaken thoroughly for 1 min. The absorbance of samples was measured under a wavelength of 450 nm using a VICTOR3™ 1420 Multi-label Counter (PerkinElmer, USA). The percent viability of cells was calculated by comparison to that of untreated control cells.

### Surface enhanced Raman spectroscopy

A home-built Raman microscope was used to record the spectral profiles from the prostate cancer sublines. A 514.5 nm Ar + Kr ion Spectra-Physics laser which excitation lines in the range from 488 to 672 nm, was used as the excitation source for the microscope. The laser was focused onto the specimen using a 1.2 NA water immersion objective lens (50×, Olympus) that also functioned to collect the backscattered signal. The collected signal was then recorded using a T64000 Jobin-Ivon Horiba triple monochromator spectrometer CCD LN_2_ cooled detector. Raman shifts down to 5 cm^–1^ and spectral resolution down to 2 cm^–1^ could be measured. The power at the sample was maintained at 1.5 mW and typical accumulation time used was 10 s. The cell: nanoparticle ratio that was used for sample preparation for the SERS experiments was similar to that of the WST-1 test. The spectra were smoothened by using 5 point FFT filter in the software Origin.

## Supplementary Material

Supplementary information
