# Supplementary material for: An impediment to random walk: trehalose microenvironment drives preferential endocytic uptake of plasmonic nanoparticles
Source: Chem Sci. 2016 Feb 23;7(6):3730–6. doi: 10.1039/c6sc00510a (PMC6013827; doi:10.1039/c6sc00510a)
Supplement: Supplementary file 1 [file SC-007-C6SC00510A-s001.pdf]

Electronic Supplementary Material (ESI) for Chemical Science.
This journal is © The Royal Society of Chemistry 2016

Supporting Information

An impediment to random walk: Trehalose microenvironment drives preferential endocytic uptake of plasmonic nanoparticles

Soumik Siddhanta,[a] Chao Zheng,[a,b] Chandrabhas Narayana,[c] Ishan Barman[a,d]*

[a]Department of Mechanical Engineering, Johns Hopkins University, Baltimore, Maryland 21218, United States.

[b]Current address: Department of Breast Surgery, Linyi People's Hospital, Linyi, Shandong 27600, China.

[c]Light Scattering Laboratory, Chemistry & Physics of Materials Unit , Jawaharlal Nehru Centre for Advanced Scientific Research, Jakkur P.O., Bangalore 560 064, India.

[d]Department of Oncology, Johns Hopkins University, Baltimore, Maryland 21287, United States.

*To whom the correspondence should be addressed. Email: ibarman@jhu.edu

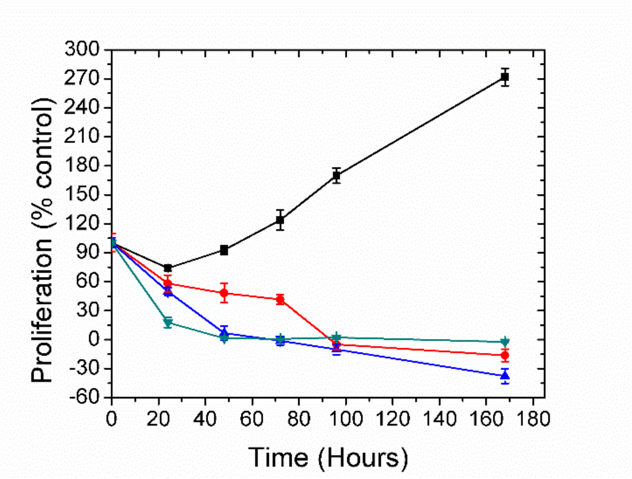

Figure S1: Cell viability by dehydrogenase activity (WST-1 test) of the PC3 FLU cells over a period of 7 days. The assays were performed with control cells (black) and cells incubated with glucose (red), nanoparticles in presence of glucose (blue) and nanoparticles in absence of glucose (green).

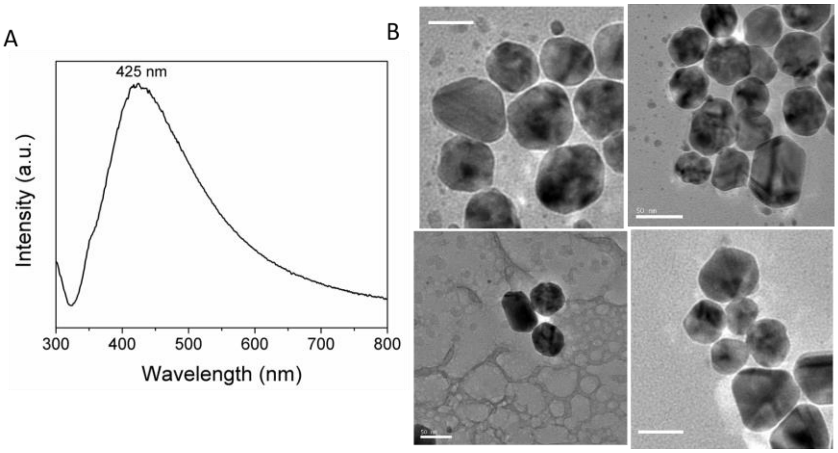

Figure S2: (A) The UV-Vis spectrum of the synthesized silver nanoparticles showing surface plasmon resonance peak at 425 nm. (B) TEM images of the synthesized nanoparticles. The nanoparticle sizes are in the range of 30-50 nm. The scale bars represents 50 nm.

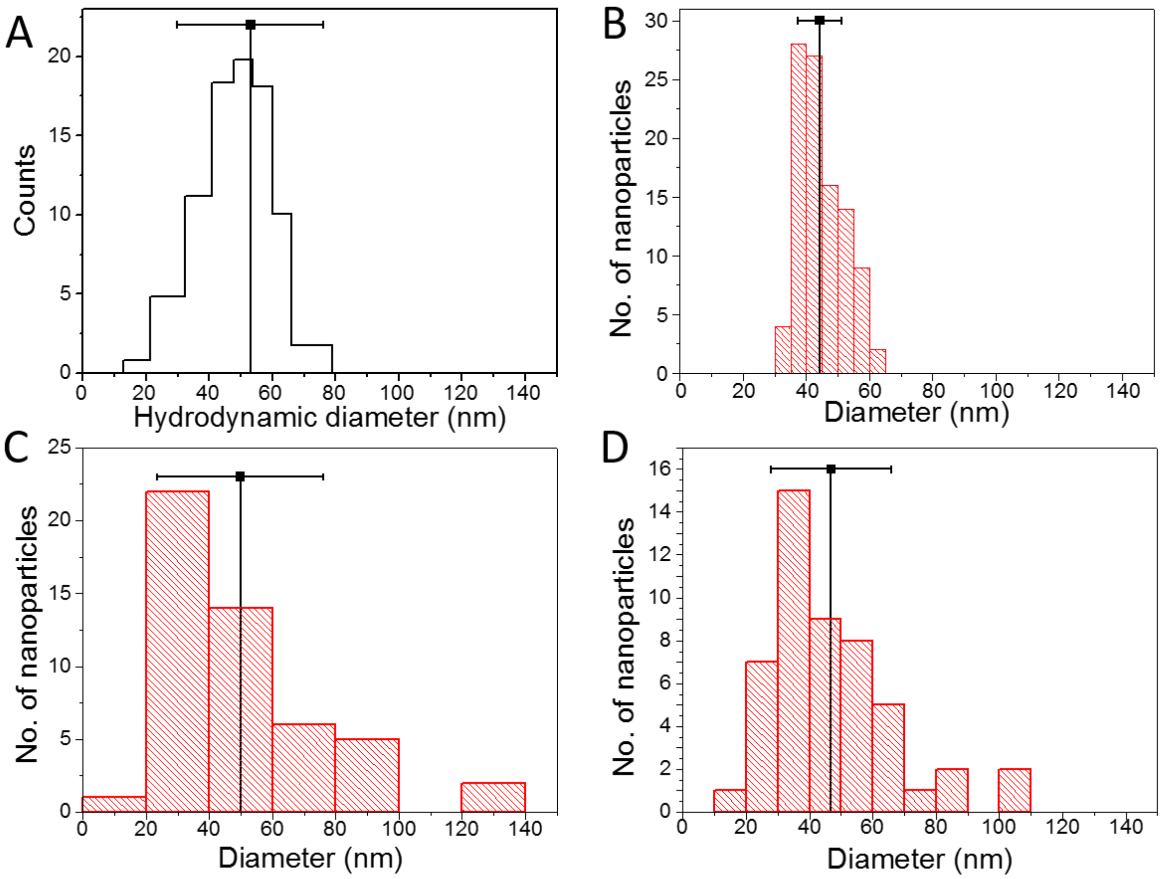

Figure S3: (A) The hydrodynamic diameter of Ag nanoparticles in solution measured by dynamic light scattering (DLS) studies. The mean and the standard deviations are 53 nm and 23 nm, respectively. (B) The diameter of Ag nanoparticles measured from TEM images. The mean and standard deviations are 44 nm and 7 nm, respectively. (C) The diameter of Ag nanoparticles that were internalized by the cells and measured from TEM images. The mean and standard deviations are 49 nm and 20 nm, respectively. (D) The diameter of Ag nanoparticles that were internalized by the cells in the presence of trehalose and measured from TEM images. The mean and the standard deviations are 46 nm and 18 nm, respectively.

Table S1: SERS band assignment of the PC3-FLU cells. The spectral band assignments were done according to available literature.[1-4]

Peak (cm^{-1})	Proposed band assignments and residues
586, 588	Symmetric vibration of phosphate
677	Ring breathing in DNA bases
750	Symmetric breathing of tryptophan
780	Cytosine/uracil, phosphodiester, phosphatidylinositol
865	Phosphate, tyrosine, polysaccharide
879	Tryptophan, C-C-N^+ symmetric stretching of lipids
999	Phenylalanine
1022	Glycogen, carbohydrate
1118, 1120	C-C stretch of lipid, O-P-O DNA backbone vibration
1247, 1249	Amide III, guanine/cytosine
1376, 1378	$\delta(CH_3)$ of lipids
1463	Lipids
1496	C=C stretch
1530	Amide II
1596	Phenylalanine
1611	Cytosine, tyrosine
1655	Amide I

References:

1. Z. Movasaghi, S. Rehman and I. U. Rehman, *Applied Spectroscopy Reviews*, 2007, **42**, 493-541.
2. K. Kneipp, A. S. Haka, H. Kneipp, K. Badizadegan, N. Yoshizawa, C. Boone, K. E. Shafer-Peltier, J. T. Motz, R. R. Dasari and M. S. Feld, *Applied Spectroscopy*, 2002, **56**, 150-154.
3. S. Siddhanta, D. Karthigeyan, P. P. Kundu, T. K. Kundu and C. Narayana, *RSC Advances*, 2013, **3**, 4221-4230.
4. M. D. Vasudevarao, P. Mizar, S. Kumari, S. Mandal, S. Siddhanta, M. M. M. Swamy, S. Kaypee, R. C. Kodihalli, A. Banerjee, C. Narayana, D. Dasgupta and T. K. Kundu, *Journal of Biological Chemistry*, 2014.
